# Deciphering neo-sex and B chromosome evolution by the draft genome of *Drosophila albomicans*

**DOI:** 10.1186/1471-2164-13-109

**Published:** 2012-03-22

**Authors:** Qi Zhou, Hong-mei Zhu, Quan-fei Huang, Li Zhao, Guo-jie Zhang, Scott W Roy, Beatriz Vicoso, Zhao-lin Xuan, Jue Ruan, Yue Zhang, Ruo-ping Zhao, Chen Ye, Xiu-qing Zhang, Jun Wang, Wen Wang, Doris Bachtrog

**Affiliations:** 1 CAS-Max Planck Junior Research Group, State Key Laboratory of Genetic Resources and Evolution, Kunming Institute of Zoology, Chinese Academy of Sciences, Kunming, Yunnan 650223, China; 2 Department of Integrative Biology, University of California, Berkeley, CA 94720, USA; 3 Beijing Genomics Institute-Shenzhen, Shenzhen 518083, China; 4 Department of Biology, Stanford University, Palo Alto, CA 94305, USA

**Keywords:** *Drosophila albomicans*, neo-sex chromosome, B chromosome

## Abstract

**Background:**

*Drosophila albomicans *is a unique model organism for studying both sex chromosome and B chromosome evolution. A pair of its autosomes comprising roughly 40% of the whole genome has fused to the ancient X and Y chromosomes only about 0.12 million years ago, thereby creating the youngest and most gene-rich neo-sex system reported to date. This species also possesses recently derived B chromosomes that show non-Mendelian inheritance and significantly influence fertility.

**Methods:**

We sequenced male flies with B chromosomes at 124.5-fold genome coverage using next-generation sequencing. To characterize neo-Y specific changes and B chromosome sequences, we also sequenced inbred female flies derived from the same strain but without B's at 28.5-fold.

**Results:**

We assembled a female genome and placed 53% of the sequence and 85% of the annotated proteins into specific chromosomes, by comparison with the 12 *Drosophila genomes*. Despite its very recent origin, the non-recombining neo-Y chromosome shows various signs of degeneration, including a significant enrichment of non-functional genes compared to the neo-X, and an excess of tandem duplications relative to other chromosomes. We also characterized a B-chromosome linked scaffold that contains an actively transcribed unit and shows sequence similarity to the subcentromeric regions of both the ancient X and the neo-X chromosome.

**Conclusions:**

Our results provide novel insights into the very early stages of sex chromosome evolution and B chromosome origination, and suggest an unprecedented connection between the births of these two systems in *D. albomicans*.

## Background

Sex chromosomes have originated independently from a pair of autosomes in a variety of species [1,2]. A striking feature shared by different systems is a degenerate, gene-poor Y/W chromosome lacking meiotic recombination. For example, the human Y chromosome only contains 78 unique protein-coding genes compared to over 1000 genes present on the X [3]. This raises the fundamental yet unresolved question of how and why the Y chromosome is degenerating after recombination is inhibited with its former homolog, the X. The Y chromosomes of most species, however, are too ancient to address this question, since only few traces of their evolutionary origin or of the processes that drove their degeneration remain (for instance, the human Y is ~170 million years old [4]). An alternative solution is to study sex chromosomes (or autosomes behaving exactly like sex chromosomes, such as 'neo-sex' chromosomes) of very recent origin [2,5,6].

**Figure 1:**
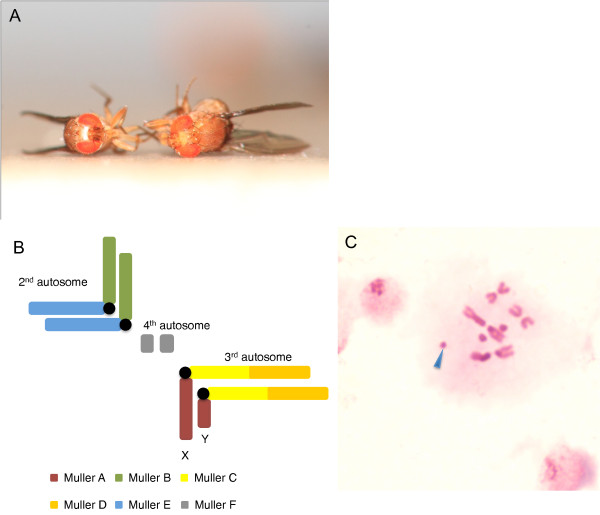
**Karyotype of Drosophila albomicans**. (**A**) *Drosophila albomicans *male (left) and female (right). Males have a silver patch on the frons. (**B**) Schematic karyotype of a male *D. albomicans*. The third autosome pair has fused to the ancestral sex chromosome pair and forms a neo-sex chromosome. Colors represent different ancestral *Drosophila *chromosome arms (Muller's elements). (**C**) B chromosomes of *D. albomicans*. The blue arrow marks a B chromosome.

*Drosophila albomicans *(Figure 1A, Additional File 1) has an extremely young neo-sex chromosome system created by the fusion of a pair of autosomes (the third chromosome, which itself is derived from the fusion of two autosomal arms, Muller's element C and D [7,8]; Figure 1B, Additional File 1) to the ancient sex chromosomes [7,9]. This karyotype resembles the formation of the human sex chromosomes, which were also created by a sex chromosome-autosome fusion [10]. Since male flies have achiasmate meiosis [11], the Y-linked autosome (the so called 'neo-Y') cannot recombine with its homolog and thus evolves like a 'true' Y. Similarly, the X-linked autosome (the 'neo-X') co-segregates with the 'true' X, and spends more time in females than males. The species' closest relative, *D. nasuta *(Additional File 1), lacks this fusion and diverged from *D. albomicans *only about 0.12 million years (MY) ago [7,9], therefore setting an upper limit to the age of the neo-sex chromosome pair. Compared to similar systems in other *Drosophila *species [12,13], the neo-sex chromosome of *D. albomicans *is the youngest reported to date and comprises almost 40% of the genome. Nearly five thousand active, newly sex-linked protein-coding genes can be used to decipher the very early evolution of sex chromosomes, and should allow us to identify signatures of selective forces of sex chromosome differentiation that have long been eroded on well-studied ancient sex chromosomes [2].

*D. albomicans *also contains recently-derived B chromosomes affecting its fertility which are absent from *D. nasuta*, thereby providing a rare opportunity to study the origin and functional significance of these bizarre chromosomes (Figure 1C and Additional File 2). B chromosomes are dispensable chromosomal elements exhibiting non-Mendelian inheritance and have been widely reported in over 1800 animal and plant taxa, but still remain an evolutionary mystery ever since their first discovery over a century ago [14]. It has been hypothesized that B chromosomes have contributed to the origin of the *Drosophila Y *chromosome [15]. Recent studies show that, in some species, B chromosomes play an important role at sex determination or accounting for antibiotic resistance and pathogenicity [16,17]. These results are in contrast to the classic view of B chromosomes as 'selfish-elements' consisting mostly of heterochromatic sequences and few functional genes. Interestingly, *D. albomicans *strains with 1-2 B's produce significantly more offspring than those with either no or more than two Bs [18]. Additionally, the frequency of B chromosomes in natural populations of *D. albomicans *shows a clear south to north geographic cline [19]. These observations suggest that some form of selection is involved in maintaining B chromosomes within the population [14,18].

Given the potential of *D. albomicans *to provide insights into these long-standing biological conundrums, we sequenced the whole genome of *D. albomicans *using Illumina's massively parallel sequencing technology. A lack of available genomes for any species in the *D. immigrans *group, to which *D. albomicans *belongs, makes a genome assembly based on reference-guided re-sequencing unfeasible. We therefore carried out a strategy combining both de novo assembly of short reads and chromosomal mapping based on a multi-species comparisons. The analysis of the resulting genome sequence allows us to address several questions including: How and to what extent has the *D. albomicans *neo-Y diverged from the neo-X within only 0.12 MY? Does the neo-Y show any signs of degeneration after such a short time scale? How did the B chromosomes originate, and can we identify any functional sequences? Finally, we explore the possibility of a direct link between the origination of the neo-sex chromosomes and B chromosomes, since both systems are absent from all species closely related to *D. albomicans *[7].

## Results and discussion

### Sequencing, assembly and annotation of the *D. albomicans *genome

We collected males from an isofemale strain containing 1-2 B chromosomes without prior purging of their polymorphisms, since inbreeding causes loss of B chromosomes. We further inbred the same strain to remove the B's, and then collected females lacking neo-Y and B chromosomes so that they can be identified by their male-specificity. We sequenced the males to high coverage (24.9 Gb, 124.5-fold genome coverage, Additional File 3) and females to moderate coverage (5.7 Gb, 28.5-fold, Additional File 3). Reads from the inbred females were used to assemble contigs (contiguous sequences without gaps derived from overlapping reads). Male data was only used for connecting the contigs into longer scaffolds without introducing any reads from the highly heterochromatic Y chromosome and possible divergent regions between the neo-sex chromosomes into the assembly (Table 1). In fact, despite having a much deeper sequencing coverage, the draft genome assembly using male reads alone has a smaller N50 length (the scaffold size above which 50% of the total length of the sequence assembly can be found, Table 1) than that derived from female reads (9.9 kb vs. 12.6 kb). We obtained 17 Mb of male-specific non-redundant sequences which lack any significant homologies to the female genome and any publicly available sequences (Methods). These regions are candidate B-linked sequences, but could also represent highly divergent regions on the Y/neo-Y chromosome, or polymorphic regions only present in the less inbred male population.

**Table 1:** Summary of the sequencing data and assembly statistics

Assembly*	Reads Used	Total Length	Scaffold N50^#^
male reads	24.9 Gb (124.5-fold)	234 Mb	9.9 kb

female reads	5.7 Gb (28.5-fold)	170 Mb	12.6 kb

reference	14.0 Gb (98.9-fold)	183 Mb	31.9 kb → 49.0 kb

* There are three versions of initial assemblies: one produced using only male reads, one using only female reads, and both of these assemblies were used to extract candidate B- or neo-Y linked sequences. The reference assembly was produced using all the female reads and part of the male reads, using parameters to maximize the N50 statistics. The reference assembly was subjected to further optimization (designated by '→' in Table 1) by comparative analysis with other *Drosophila *genomes and gene annotations. ^# ^N50: the length *L *where 50% of all nucleotides in the assembly are contained in contigs/scaffolds of size ≥ *L*.

The availability of 12 *Drosophila genomes *helps us to optimize the assembly. We inferred that conserved syntenic protein-coding gene pairs whose order and orientation are the same among all the 12 *Drosophila genomes *should have experienced few rearrangements in the *D. albomicans *genome. We then collected scaffolds that aligned to the same gene or to regions consisting of conserved syntenic gene pairs [20], and merged 1,433 scaffolds into 701 super-scaffolds. The final female reference genome assembly of *D. albomicans *has an improved scaffold N50 from 31.9 kb to 49.0 kb (Table 1). To validate the assembly accuracy, we designed primer pairs for 154 fragments randomly selected throughout the genome, about half of which span at least one gap region. 148 pairs (96%) successfully produced unique PCR products with expected lengths, with the remaining 4% all spanning a gap region that may be too long to be amplified. We also aligned the assembly against 820 EST sequences derived from a male of another strain of *D. albomicans *[9], and 88.5% of ESTs aligned to the genome assembly with an overall identity of 98.8%. Contamination is low: only 289 scaffolds, comprising 739 kb (0.3% of the total sequence) showed evidence for human or bacterial sources. These results show that we have produced a high-quality draft assembly of the *D. albomicans *genome.

We annotated 14,042 protein-coding genes on non-B scaffolds and 272 genes in male-specific scaffolds (and thus candidate B-linked genes), using both *de novo *gene prediction and homology-based methods. We found that 5.5% of the *D. albomicans *genome consists of repetitive elements (compared to 5.35% of *D. melanogaster *[21], Additional File 4), suggesting that our short-read assembly has captured a sizeable fraction of repeat sequences. Finally, previous analysis of 12 *Drosophila *genomes has shown that 95% of genes exhibit no movement between chromosome arms (called 'Muller elements' in *Drosophila species*) across species [20]. This allowed us to assign 11,949 (85.1%) D. *albomicans *protein-coding genes or 5576 scaffolds (109 Mb, 52.8%) into chromosome arms based on their alignments with the *D. virilis *genome.

### Genomic divergence between the neo-sex chromosomes

To investigate the divergence between the neo-sex chromosomes after recombination ceased, we mapped male and female reads separately to the female genome and characterized the level of variation for each chromosome. SNP and short indel (< 6 bp) densities calculated from female reads against the neo-X reference reflect segregating variation among different neo-X chromosomes, whereas those derived from the less inbred males reflect both neo-X/Y divergence and segregating variation among different neo-X chromosomes. Sequence coverage is similar across chromosomal arms and thus should not bias comparisons across chromosomes (Additional File 5).

**Figure 2:**
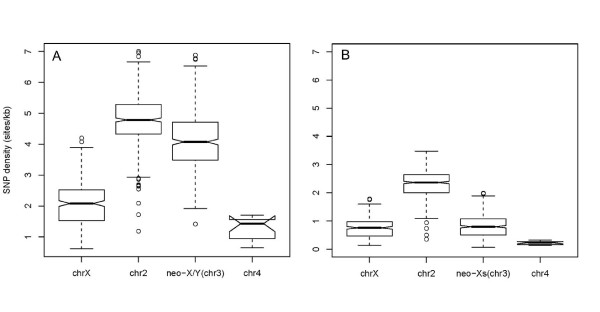
**Patterns of heterozygosity across chromosomes**. SNP densities along each chromosome were calculated within a 1 Mb window with a step size of 100 kb. (**A**) SNP densities calculated based on mapping of male reads. (**B**) SNP densities calculated based on mapping results of female reads.

Females generally show lower levels of variation relative to males (Figure 2, Additional File 6 and 7), consistent with the more severe inbreeding of the females used for sequencing. The neo-X reveals a similarly low level of SNP density as the ancient X chromosome relative to autosomes, either due to the bottleneck effect of inbreeding or very recent selective sweeps [22,23]. In contrast, SNP density of the neo-sex chromosome calculated from male data is much higher than that of the ancient X (*P *< 10^-3^, Chi-square test, Table 2, Figure 2A). Elevated SNP density levels on the neo-sex chromosomes of males can mainly be attributed to sequence divergence between the neo-X and neo-Y chromosome. Indeed, the ratio of SNP density in males versus females is significantly higher for the neo-sex chromosomes (which in males includes both segregating neo-X variants and neo-X/neo-Y divergence) relative to the ancient X and the autosomes (Table 2, Fisher's exact test, P < 0.01). Fixed neo-X/neo-Y differences are necessarily present in the male reads pool at a roughly 1:1 ratio whereas segregating variation on either chromosome may be found at a variety of frequencies. Consistent with many neo-sex-linked SNPs in males reflecting fixed differences between the neo-X and neo-Y alleles, the fraction and density of SNPs present at a ratio around 1 is the highest on the neo-sex chromosomes in males (17.7% and 0.7 sites/kb; Additional File 8). Thus, these data demonstrate that the neo-X/neo-Y chromosome pair has already started to diverge within only 0.12 MY. However, the non-recombining fourth chromosome shows similarly increased levels of diversity in males relative to females, indicating that some sampling effects on chromosome-specific differences in levels of diversity between the sexes cannot be ruled out. Interestingly, although both neo-X polymorphisms and neo-X/neo-Y divergence contribute to the overall SNP density of the neo-sex chromosomes in males, it is still lower than that of autosome 2 (Figure 2A, but not within coding regions, see below). This could be explained by longer coalescent times for autosomes than neo-sex chromosomes, or by demographic factors or selective sweeps reducing neo-X diversity as mentioned above. Also, excessive inversion polymorphisms of autosome 2 (see [24] and also Additional File 9) could result in a higher overall SNP density [25]. It will be of great interest to study patterns of diversity of the various chromosomes in more detail using a larger population sample.

**Table 2:** Comparison of neo-sex chromosomes with other chromosomes

Chromosome	chrX (Muller A)	neo-sex (Muller C + D)	chr2 (Muller B + E)	chr4 (Muller F)^†^
exonic SNP density *(♂/♀)	2.04/1.01(2.02)	4.61/0.97(4.75)	4.35/3.33(1.30)	0.18/0.01(18)

intronic SNPs density *(♂/♀)	2.58/0.77(3.35)	5.57/0.86(6.47)	5.84/2.75(2.12)	0.99/0.03(33)

putative pseudogenes No./total^#^	11/2055	80/4751	54/5056	1/87

*Ka *average (× 10^3^)^§^	1.03/0.42	2.02/0.58	1.89/1.26	0.73/0.16

*Ka *sd. (× 10^3^)	9.48/4.56	19.56/6.82	18.11/14.3	29.13/10.87

Tandem Duplications (%)^||^	13.8/5.6	20.7/5.5	15.3/3.79	14.9/5.8

* SNP density (sites/kb) was calculated using exon or intron regions of scaffolds mapping to the respective chromosomes, and shown as SNP density in male/SNP density in female, and their ratio in parentheses.

^# ^Male-specific putative pseudogenes were identified by premature stop codons, frameshifts or mutations at start codons. Shown are the pseudogene number/total mapped genes on each chromosome.

^§ ^Shown are nonsynonymous substitutions calculated in male/female. Sd.: standard deviation.

^|| ^Shown are proportions of tandem duplications among all the SV events for each chromosome identified in male/female.

^†^Due to the low number of genes on chr4, inferences on this chromosome may be strongly affected by sampling effects.

### Early degeneration of neo-Y coding regions

The divergence between the neo-X and neo-Y is likely caused by the accumulation of deleterious mutations on the neo-Y after the inhibition of homologous recombination [2]. To investigate this process, we compared patterns of SNP densities in the coding regions of neo-X- and neo-Y-linked sequences. We found that the male to female ratio of exonic SNP density is higher on neo-sex chromosomes than on any other chromosome (Table 2), suggesting an excess of mutations accumulating in coding regions of neo-Y-linked genes. To evaluate their deleterious effects, we characterized putative pseudogenes on the neo-Y chromosome whose coding regions contain premature stop codons or frame-shift mutations. We first identified ancestral pseudogenes (i.e. those shared between the neo-X and neo-Y chromosome) by mapping female reads to the reference genome and keeping only those genes that were not found to be pseudogenized in the female data. After applying the same procedure to all chromosomes, we identified a total of 80 putative pseudogenes that mapped to the neo-sex chromosome in males, a significantly higher fraction than on any other chromosome (*P *< 0.05, Fisher's exact test, Table 2). These pseudogenized presumably neo-Y-linked alleles did not show any significant enrichment for specific gene ontology (GO) terms, suggesting that genes are inactivated randomly during early neo-Y degeneration [5]. Additionally, the neo-sex chromosome genes show slightly higher levels of pairwise nonsynonymous diversity (Table 2) and slightly relaxed constraint on synonymous sites (as measured by codon usage indices; Additional File 10).

Together, these data suggest that deleterious mutations have already begun to accumulate at some neo-Y protein-coding genes. The proportion of pseudogenized neo-Y loci in *D. albomicans *(2% of total neo-X loci in 0.12 MY) is much lower than that in *D. miranda *(46% of genes have become non-functional [5]), a species where a neo-sex chromosome originated about 1 MY ago. The neo-X chromosome of *D. miranda *shows partial dosage compensation [26], which can render some genes on the neo-Y functionally redundant and speed up Y degeneration of genes that are dosage compensated on the neo-X. Also, the rate of degeneration is expected to be highly non-uniform over the course of Y evolution, and different evolutionary processes causing mutation accumulation show different temporal dynamics [27]. In particular, Muller's ratchet and background selection are more likely to dominate degeneration of Y chromosomes at very early stages, such as that of *D. albomicans*, while genetic hitchhiking of deleterious mutations together with beneficial alleles is more likely to contribute to Y degeneration at later stages [27]. It will be of great interest to test the role of these evolutionary models of Y degeneration using population genomic studies of the *D. albomicans *neo-sex chromosomes.

### The neo-Y has an excess of tandem duplications

We also investigated structural variants (SVs) in the *D. albomicans *genome, including duplications, inversions and large-scale insertion/deletion events (> 150 bp). To identify SVs, we took advantage of abnormally aligned read mate-pairs mapped against our reference genome. For example, sequence reads from the same insert are supposed to have opposite orientations when aligned to the reference genome. However, reads surrounding the breakpoint of tandem duplications would instead face the same orientation [28] (Additional File 11). Since females are more inbred and were used for the initial genome assembly, many fewer reads derived from females showed mate-pair violations (31.8% of male pairs versus only 4.5% of female pairs).

**Figure 3:**
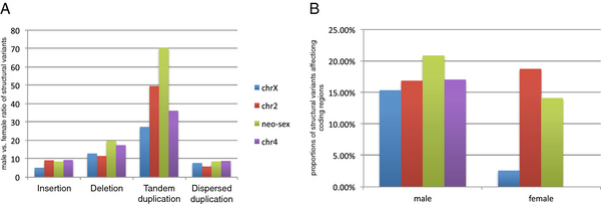
**Structural variation in the *D. albomcians *genome**. (**A**) The male to female ratio of different structural variants (insertions, deletions, tandem duplication, dispersed duplications) on each chromosome. (**B**) The proportion of tandem duplications overlapping with protein-coding regions on each chromosome for males and females (no tandem duplications on the fourth chromosome overlapping coding regions were observed in females).

By characterizing all the abnormally aligned mate-pairs against the reference genome, we detected a total of 86,890 SVs (each SV event is supported by at least 3 mate-pairs, see Methods). We found deletions to be more common than insertions on all chromosomes (50% to 70.3% SV sites, Additional File 9 and 12), consistent with a previous report in other *Drosophila *species [29]. Intriguingly, we found the male-to-female ratio of tandem duplications to be highest on the neo-sex chromosome among all the chromosomes and types of SVs investigated (Figure 3A and Additional File 9), suggesting an accumulation of tandem duplications on the neo-Y. The lack of recombination on the neo-Y implies that it is mechanistically harder to remove pre-existing duplicated sequences through a recombination event. In addition, reduced efficiency of purifying selection on the neo-Y could lead to less efficient elimination of slightly deleterious duplicated sequences. Notably, neo-Y chromosomes have the highest proportion of tandem duplicates overlapping with coding regions, supporting the notion that slightly deleterious effects of extra gene or exon doses associated with these tandem duplications are not being effectively purged from the neo-Y (Figure 3B). Interestingly, we found no bias in the chromosomal distribution of dispersed duplications (Additional File 13). Unlike for tandem duplications, the removal of dispersed duplications probably does not rely on homologous recombination between alleles, and thus their distribution may be less influenced by differences in the recombination environments of the neo-sex chromosomes.

### Origin and evolution of B chromosomes

**Figure 4:**
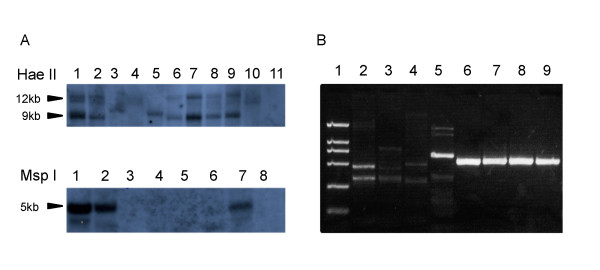
**B-specific sequences of *D. albomicans***. (**A**) Validation of the identified B-linked probe with Southern Blotting. Up panel: Genomic DNAs digested with *HaeII*. Lanes 3-5,10 show hybridization results with genomic DNA from *D. albomicans *strains without B's. Lane 11 is the negative control lane using genomic DNA of *D. melanogaster*. Other lanes show hybridizations with DNA from *D. albomicans *strains containing B's. Arrows indicate differential bands between B and no-B strains. Down panel: Genomic DNAs digested with *MspI*. Lanes 3-6 show *D. albomicans *strains without B's. Lane 8 is the negative control and other lanes are *D. albomicans *strains with B's. (**B**) PCR assays using B-chromosome derived primers. Lane 2-5 show PCR products obtained from *D. albomicans *strains without B's, and lanes 6-9 are from strains with B's. Lane 1 is the DL2000 marker.

To understand the genomic composition and functional significance of B chromosomes in *D. albomicans*, we attempted to isolate B chromosome sequences and identify B-linked genes. We first randomly selected scaffolds from the 17 Mb of male-specific sequence and performed PCR amplification using females of different *D. albomicans *strains with and without Bs. However, none of the 25 scaffolds tested showed clear linkage to B chromosomes, suggesting that B chromosomes either largely share sequences with standard chromosomes or that the identified scaffolds are either male-specific (neo-Y or Y-linked), located in heterochromatic sequences that could not be mapped or assembled with the lower-coverage female data, or represent polymorphisms segregating in the less inbred male data. We thus took advantage of a previously identified 260 bp B-linked fragment, which was cloned using subtractive hybridization (W.W., unpublished data, Figure 4A), to bait B-specific sequences. Four scaffolds showed high levels of sequence similarity to this probe, with scaffold S51440 giving the best match (Additional File 14). To validate the assembly of this scaffold and confirm that it is indeed B-specific, we designed ten primer pairs spanning and surrounding the aligned region on this scaffold. One pair of primers amplified a fragment of the expected length in all B-containing isofemale strains but not in strains lacking B chromosomes (in a total of 8 lines tested), thereby confirming that the amplified sequence is B-specific (Figure 4B). All other primer pairs amplified products in both B and non-B strains, indicating that this scaffold is largely composed of sequences highly similar to standard chromosomes. This high level of sequence homology would explain why our genomic approach failed to identify B-linked sequences based on presence/absence of DNA scaffolds in samples with and without B chromosomes. Interestingly, we were able to successfully detect transcription from this B-linked fragment using an RT-PCR assay. This fragment carries a transcribing unit homologous to the gene *shg *on chr2R and contains a diagnostic SNP in all the four B-containing strains but not in the four non-B strains, implying that functional genes may exist on this B chromosome.

Intriguingly, almost all the sequences of the four scaffolds that show homology to the B-derived sequence are homologous to regions that are located on the ancient X and/or the neo-X chromosome of *D. albomicans*, and the scaffold with the highest sequence similarity to the B-specific probe (scaffold S51440) contains sequence mapping to both chromosomes (Additional File 15). Notably, different portions of S51440 mapped to sub-centromeric and sub-telomeric regions of the ancient X and neo-sex chromosomes, respectively, and this mapping holds across a range of *Drosophila species *(Additional File 15). In addition, S51440 shows three-fold elevation in simple repeat content compared to the rest of the genome (20.86% versus 6.69%; Table S2). We also aligned male reads containing B sequences and female reads without B sequences to the reported Drosophila centromeric sequences [30]. The proportion of male reads that align to these centromeric sequences is much higher than that of female reads (1.2 × 10^-5 ^vs. 3.2 × 10^-6^), although it is unclear how much of this can be attributed to sequences contained in the neo-Y or the ancestral Y chromosome. Taken together, these characteristics of candidate B-linked sequences suggest that *D. albomicans *B chromosomes may have originated as a byproduct from (sub-)centromeric/telomeric fragments created by the fusion of the ancient third autosome and the ancestral sex chromosomes. Consistent with an association between the formation of B and neo-sex chromosomes, all close relatives of *D. albomicans *within the *D. immigrans *species group lack the 'neo-sex' fusion [7,9] as well as B chromosomes. B chromosomes may have subsequently acquired some unique sequences or genes after their origination by mutations or rearrangements.

These insights allow us to further develop a model of neo-sex and B chromosome evolution in *D. albomicans*. It has been hypothesized that centromere-fused chromosomes may be favored during asymmetrical female meiosis if the excess of centromeric satellites facilitates their entry into oocytes [31,32]. Such a selective advantage would apply to the neo-X fusion, i.e., the fusion of the third autosome (chr3) to the ancestral X chromosome (chrX) but not the male-limited neo-Y. Indeed, the neo-X chromosome remains polymorphic within experimental hybrid populations from female *D. albomicans *and male *D. nasuta*, which contain both the fused chrX and chr3 (i.e. the neo-X) and unfused chrX and chr3 (i.e. the ancestral conditions), but no neo-Y chromosomes [33]. Further, the neo-X can increase in frequency in such a hybrid population in a background with neo-Y chromosomes [33]. In contrast, a polymorphic neo-Y is excluded within a few generations in hybrids without the neo-X fusion. The formation of the neo-X could result in centromeric imbalances between paired chromosomes in males [34], which could have been remedied by a second fusion producing the neo-Y [33] or by loss of centromeric fragments of the neo-X. Indeed, a polymorphic neo-Y can reach fixation within a few generations in hybrids containing the neo-X [33]. The lost centromeric fragments from these fusions could have given rise to the B chromosomes found in *D. albomicans*. This, of course, does not exclude the possibility that genes or genomic regions derived from other chromosomes also contributed to the formation of B chromosomes, and they may be maintained within the population because of newly acquired functions. This might explain the fact that *D. albomicans *strains with 1-2 B's produce significantly more offsprings than strains with no or more B chromosomes (Additional File 16) [18]. Further identification of B-linked scaffolds and the genes they carry will help to understand the phenotypic effects of B chromosomes in *D. albomicans*.

## Conclusions

We provide a draft genome sequence and evolutionary analyses of *D. albomicans*, a species that contains extremely young neo-sex chromosomes and B chromosomes, to address questions on the origination and evolution of these chromosomal systems. Genomic comparison between males and females reveals that the neo-Y chromosome has already begun to accumulate an excess of deleterious mutations, including nonsense mutations that presumably caused some genes to become non-functional. We also found that the neo-Y chromosome harbors an excess of tandem duplications. Comparative analysis also identified a B-linked sequence that is homologous to regions of both the neo-X and ancient X chromosome. This result is consistent with the idea that B chromosomes originated from the standard chromosomes as a product of the sex chromosome - autosome fusion. Functional characterization of the transcribing unit located on the B chromosome and future studies of other B-linked genes should help to explain the fertility effects of these dispensable chromosomal elements in *D. albomicans*.

## Methods

### Fly strains and karyotyping

We collected *D. albomicans *individuals from the wild in Kunming, China, and established isofemale strains for genome sequencing. We karyotyped a total of 15 strains following the method described in [35]. In brief, ganglions were dissected from third stage instar larvae and treated with 0.065 mg/ml of colchicine solution, followed by hypotonic treatment with 0.075 mol/l KCL solution. After fixation by Carnoy solution, we dried the slides and stained them with Giemsa solution (Fluka) for 1.5 h and then washed them in water. Metaphase images were then captured under a microscope (Nikon). B-chromosome number in the Kunming *D. albomicans *population ranges from 0 to 7 (Additional File 2). We selected one strain, KM55-5, containing 1-2Bs in about 70% investigated karyotypes, since carrying 1-2 Bs has been shown to maximize offspring number in *D. albomicans *[18]. The strain was karyotyped every 2 generations to assure B-chromosome maintenance, and reared for 13 generations before DNA extraction. We picked around 40 males and extracted their genomic DNA with a PUREGENE DNA Isolation Kit (Gentra System). To screen individuals without Bs from this strain, we inbred pairs of one single male and one virgin female for four generations and karyotyped their offspring (at least 20 larvae).

We established a primary cell line using early stage embryos (4-6 h) of the KM55-5 strain to estimate the genome size of *D. albomicans *[36]. About 5 × 10^5 ^cells were harvested and washed twice with PBS solution. Cells were then transferred to FACS tubes kept on ice, and 50 μl RNase A (1 mg/ml) and 100 μl propidium iodide (PI, 1 mg/ml) were added to each sample. Samples were analyzed on a FACScan flow cytometer (Becton Dickinson). The result was then compared with chicken red blood cells and *Drosophila *S2 cells which were prepared at the same time.

### Sequencing and assembling

Solexa library preparation and sequencing followed the manufacturer's standard instructions (Illumina). Briefly, we prepared 5 ug genomic DNA from outbred virgin B-containing males and inbred virgin females without Bs, shearing them with Nebulizer (Invitrogen). Paired-end libraries were prepared and subject to Solexa sequencing, with read sequencing from both ends. Lanes yielding an abnormal GC content or read bases with low Q20 length (< 30 bp) were removed from the raw data before assembly or mapping. We used EST sequences generated from a cDNA library derived from males of another *D. albomicans *strain (strain 15112-1751.4) [9] for assembly quality assessment.

We used SOAPdenovo http://soap.genomics.org.cn/[37] and tried different *k*-mer parameters and combinations of read lanes of male and female reads. Female reads, although adding up to a much lower genomic coverage than male reads (28.5 fold vs. 124.5 fold), consistently produced a higher contig and scaffold N50 size under the same assembly parameters. This suggests that sequence coverage of 30-fold is sufficient to produce a decent-quality assembly, and that male-limited neo-Y or Y-linked sequences or B chromosomes deteriorate the assemblies. We therefore assembled the reference genome using all the female reads (28.5-fold) to construct contigs, and part of the male reads (70.4-fold, selected according to their read qualities) to link contigs into scaffolds (these were not used in the contig assembly to prevent the introduction of sex-linked and B-linked heterozygous sites that would deteriorate the quality of the assembly). Short reads were then mapped back to the scaffolds to fill in gaps. All of the assemblies used a *k*-mer value of 31, and the low quality first and last 5 bp of all reads were trimmed. Scaffolds assembled solely from male or female reads were also produced to extract candidate B-linked sequences (male-specific sequences that were not part of the ancestral Y). We aligned scaffolds assembled from male reads only to those assembled from female reads and selected male-specific scaffolds as candidate B regions using a blast E-value cutoff of 10^-5 ^and a maximum GenBlastA alignment span size of 25% of the query [38]. A total of 63 Mb of male scaffolds, for which less than 20% of their length could be aligned to female scaffolds were considered as candidate B scaffolds.

To further optimize the *D. albomcians *assembly, we blasted [39] all the *D. virilis *proteins ftp://flybase.net/genomes/Drosophila_virilis/current/fasta/ against our raw assembly, using an *E*-value cutoff of 10^-3^. When several scaffolds that aligned to a *D. virilis *protein overlapped with each other, only the scaffold with the highest alignment score was kept. We then merged scaffolds into larger ones according to their order along the query gene. We also extracted *D. virilis *proteins that have syntenic relationship, which are conserved across other 11 *Drosophila *species [20]. *D. virilis *scaffolds encompassing these proteins were concatenated into superscaffolds as reference. We then aligned exon-level merged *D. albomicans *scaffolds with sensitive parameters (- = r 1 -q -1 -G 1 -E 2 -W 9 -F "m D" -U -e 1e-5) to *D. virilis *sequences and only retained hits spanning over 50% of the query. Aligned scaffolds were ordered and concatenated with 500 interval Ns.

### Annotations

We downloaded the protein sequence annotations of 8 *Drosophila *species (all species available on ftp://ftp.flybase.net/releases/ excluding *D. sechellia, D. simulans, D. persimilis *and *D. erecta *because their sister species were used instead). We removed redundant proteins (proteins that share orthologous relationships) and our final set of annotation queries consisted of 66,877 proteins that were blasted against the *D. albomicans *mosaic scaffolds. The top two query proteins (ranked by alignment span sizes) were chosen to represent *D. albomicans *loci and fed to GeneWise [40], in order to determine their gene structures. GeneID and SNAP [41,42] were used to check the GeneWise predictions and results were reconciled by GLEAN [43]. We only retained proteins on non-B sequences that have more than 50 amino acids and were supported by both GeneWise and GeneID/SNAP. For candidate B sequences, we did not require the predicted proteins to be supported by GeneWise. All the proteins and scaffolds were then subjected to a blast search against the NCBI nt/nr database ftp://ftp.ncbi.nih.gov/blast/db/20090216/. We removed proteins/scaffolds showing high sequence similarities (at least 50% sequence identity over 80% length) with human or bacteria sequences. We also narrowed down the candidate B scaffolds, assuming that B chromosomes would not show sequence similarities with known sequences, and removed all candidate B scaffolds that had protein-coding sequences annotated that showed homology to any published sequences. After this screening procedure, we retained a total of 17 Mb candidate B sequences. We annotated repetitive elements (Table S2) using two well-established libraries built from the 12 *Drosophila genomes*, one from Repbase and one from ReAS ftp://ftp.genomics.org.cn/pub/reas/drosophila/[44,45] by RepeatMasker http://www.repeatmasker.org/. We also annotated simple tandem repeated sequences using the Tandem Repeat Finder (Table S2) [46].

We ordered and oriented *D. virilis *scaffolds to build chromosomal sequences based on the results of [47]. We established reciprocally-best orthologous relationship between predicted *D. albomicans *and *D. virilis *proteins ftp://ftp.flybase.net/releases/. We also aligned *D. albomicans *scaffolds with *D. virilis *chromosomes by ranking the ratio of identical bases to aligned span size and picked the highest ranked scaffold to assign *D. albomicans *proteins to different chromosomes.

### Identifications of SNP, indel and structural variation

We used the Short Oligonucleotide Alignment Program (SOAP) [48] to separately align male and female reads back to the genome assembly using the 'paired-end' mode and allowing for a maximum of 2 nucleotide mismatches per read. Using these parameters, reads were only aligned when their relative orientations are correct and their inferred distance along the assembly (the aligned span size) was consistent with the estimated library insert size. We then identified SNPs using SOAPsnp http://soap.genomics.org.cn/soapsnp.html, which showed a 99% consistency for SNP identification compared with Illumina HapMap 1 M BeadChip Duo data [49]. SOAPsnp identifies SNPs based on Bayes' theorem, taking into account both sequencing and alignment qualities. Heterozygosity levels for each chromosome were then calculated using customized Perl scripts.

We further aligned reads using the 'single-end' mode to identify potential short indels and structural variation. Reads that failed to align before when indels were not permitted were re-aligned and allowing for 1-6 bp short indels (which accounted for more than 80% of short indels in the *Drosophila melanogaster *genome [50]). Only short indels with at least one supporting read from each strand were retained. To identify structural variation, we utilized aligned read pairs with abnormal span size or orientation (i.e. those showing mate-pair violations) using SOAPsv http://soap.genomics.org.cn/. The experimentally estimated standard deviation of the library insert size from gels is about 50 bp. Long insertion/deletion events were inferred when the distance between the aligned forward and reverse read pairs was larger or shorter than the library insert size by three times the standard deviation. Tandem duplications were called when a pair of reads was aligned as 'reverse-forward' rather than the normal 'forward-reverse' orientation from 5' to 3' along the reference assembly sequence. At least three supporting abnormally aligned reads pairs were required for a structural variant to be kept in our analysis.

### Evolutionary analysis

To discriminate neo-X and neo-Y alleles, we first identified SNP or indel sites derived from female reads. We introduced these alleles into the reference scaffolds that mapped to the to neo-sex chromosomes, assuming they represented the neo-X alleles. We then modified these scaffolds by introducing alternative nucleotides and indels found only in males to obtain the homologous neo-Y alleles. We ran GeneWise [40] on the respective allelic sequences and characterized premature stop codons and frameshift mutations at neo-Y-linked genes. GeneWise results indicating different start positions for neo-X and neo-Y coding sequences were checked manually to ensure that such differences were caused by mutations at start codons and not alignment artifacts. Similarly, we characterized putative pseudogenes on the neo-X chromosome based on female reads only. These were taken as ancestral pseudogenes that already existed before the origin of the neo-sex chromosomes, and were thus removed from the analysis.

Since there are no close outgroup sequences available, we could not compare separately substitution rates on the neo-X and the neo-Y chromosome; we therefore compared the rates between the neo-X and neo-Y alleles. We used annotated protein sequences and GeneWise to align homologous neo-sex genes codon by codon. Synonymous and nonsynonymous substitution rates were calculated with the KaKs_calculator [51] using the method of Yang and Nielson [52]. We measured codon bias of neo-X and neo-Y alleles using the codonW http://sourceforge.net/projects/codonw/ and cai programs within the EMBOSS package http://emboss.sourceforge.net/. Different parameters, including effective number of codons (ENC), codon bias index (CBI), frequency of optimal codons index (FOP) and codon adaptation index (CAI) consistently showed a very weak relaxation of selective constraints at synonymous sites of neo-Y-linked genes.

### B-linked sequence analysis

We first randomly chose 25 candidate B-linked scaffolds that were identified computationally and designed 100 PCR primer pairs to validate their B-chromosome linkage using genomic DNA of *D. albomicans *strains with and without B's. None of the primer pairs discriminated between strains with and without B's. We therefore identified scaffolds that had previously been mapped to the B-chromosome by cloning through subtractive hybridization (W.W unpublished). To confirm B-linkage of these sequences, we extracted genomic DNA with PUREGENE DNA Isolation Kit (Gentra) from fly strains with and without B's. Genomic DNA was digested with restriction enzymes, separated on agarose gels and transferred to nylon membranes (Roche Molecular Biochemicals) by Southern blotting. DIG-labeled probes were hybridized to the membrane to confirm the presence/absence of B-sequence in different strains.

We computationally queried the probe sequence against all D. albomicans scaffolds and identified four scaffolds with high sequence similarity to the B-linked sequence. We performed PCR experiments using primers spanning both the probe sequence and the sequence adjacent to it on the candidate scaffolds to exclude assembly artifacts. We also extracted total RNA from *D. albomicans *adults with and without B's using an RNAeasy Mini Kit (Qiagen) and performed RT-PCR for predicted genes on the B-linked scaffolds. We performed blast searches using these candidate B scaffolds against the genome sequence of other *Drosophila species *(with searching parameters of relatively low stringency; -r 1 -q -1 -G 1 -E 2 -W 9 -F "m D" -U -e 1e-5, as these species have diverged from D. albomicans more than 40 MY ago [53]). Chromosomal mapping information for the resulting scaffolds in these other species was then retrieved through the UCSC Genome Browser based on their blastz comparison with the *D. melanogaster *genome [54,55]. We also downloaded centromeric sequences of *Drosophila *[30] and mapped male and female reads separately using SOAP.

## Competing interests

The authors declare that they have no competing interests.

## Authors' contributions

WW, JW, JZ and QZ designed the study. QZ, WW, JW, SR, BV and DB wrote the manuscript. LZ, YZ and RZ collected the samples. ZX, XZ, BM, JM, QZ, JL, YL, ZL constructed the pair-end libraries and conducted the Illumina sequencing. QZ, HZ, QH, GZ, JR, RL and DB performed the data analyses. QZ and LZ performed the experimental validations. Correspondence and requests for materials should be addressed to DB (dbachtrog@berkeley.edu), WW (wwang@mail.kiz.ac.cn) or JW (wangj@genomics.org.cn). All authors read and approved the final manuscript.

## Supplementary Material

Additional file 1 **Figure S1 Karyotyp of *D. albomicans *and other *Drosophila *species**. Click here for file

Additional file 2 **Figure S2 B chromosomes of *D. albomicans***. Click here for file

Additional file 3 **Table S1 Data summary of Illumina sequencing**. Click here for file

Additional file 4 **Table S2 Repetitive elements in the *D. albomicans *genome**. Click here for file

Additional file 5 **Table S3 Sequence coverage comparison among chromosomes**. Click here for file

Additional file 6 **Figure S3 Short insertion (1-6 bp) densities along each chromosome**. Click here for file

Additional file 7 **Figure S4 Short deletion densities along each chromosome**. Click here for file

Additional file 8 **Table S4 SNP statistics for each chromosome**. Click here for file

Additional file 9 **Table S6 Structural variation identified by abnormally mapped male read pairs**. Click here for file

Additional file 10 **Table S5 Codon usage on neo-sex chromosomes**. Click here for file

Additional file 11 **Figure S5 Identification of structural variation using mate-pair information**. Click here for file

Additional file 12 **Figure S6 Fraction of different SV on Each Chromosome**. Click here for file

Additional file 13 **Figure S7**. Proportions of dispersed duplications overlapping coding regions on each chromosome. Click here for file

Additional file 14 **Table S7 Statistics of scaffolds homologous to the B-specific probe**. Click here for file

Additional file 15 **Table S8 Mapping result of candidate B-linked scaffolds with *D. grimshawi *and *D. mojavensis***. Click here for file

Additional file 16 **Table S9 Comparison of number of progenies in different *D. albomicans *strains**. Click here for file

## Accession Numbers

Short pair-end reads from this study have been deposited in the NCBI Sequence Read Archive (SRA) under the accession number SRA009775 and BioProject ID PRJNA81745.
