# Supplementary material for: Deciphering neo-sex and B chromosome evolution by the draft genome of Drosophila albomicans
Source: BMC Genomics. 2012 Mar 22;13:109. doi: 10.1186/1471-2164-13-109 (PMC3353239; doi:10.1186/1471-2164-13-109)
Supplement: Additional file 1 — Figure S1 Karyotyp of D. albomicans and other Drosophila species. [file 1471-2164-13-109-S1.DOCX]

**
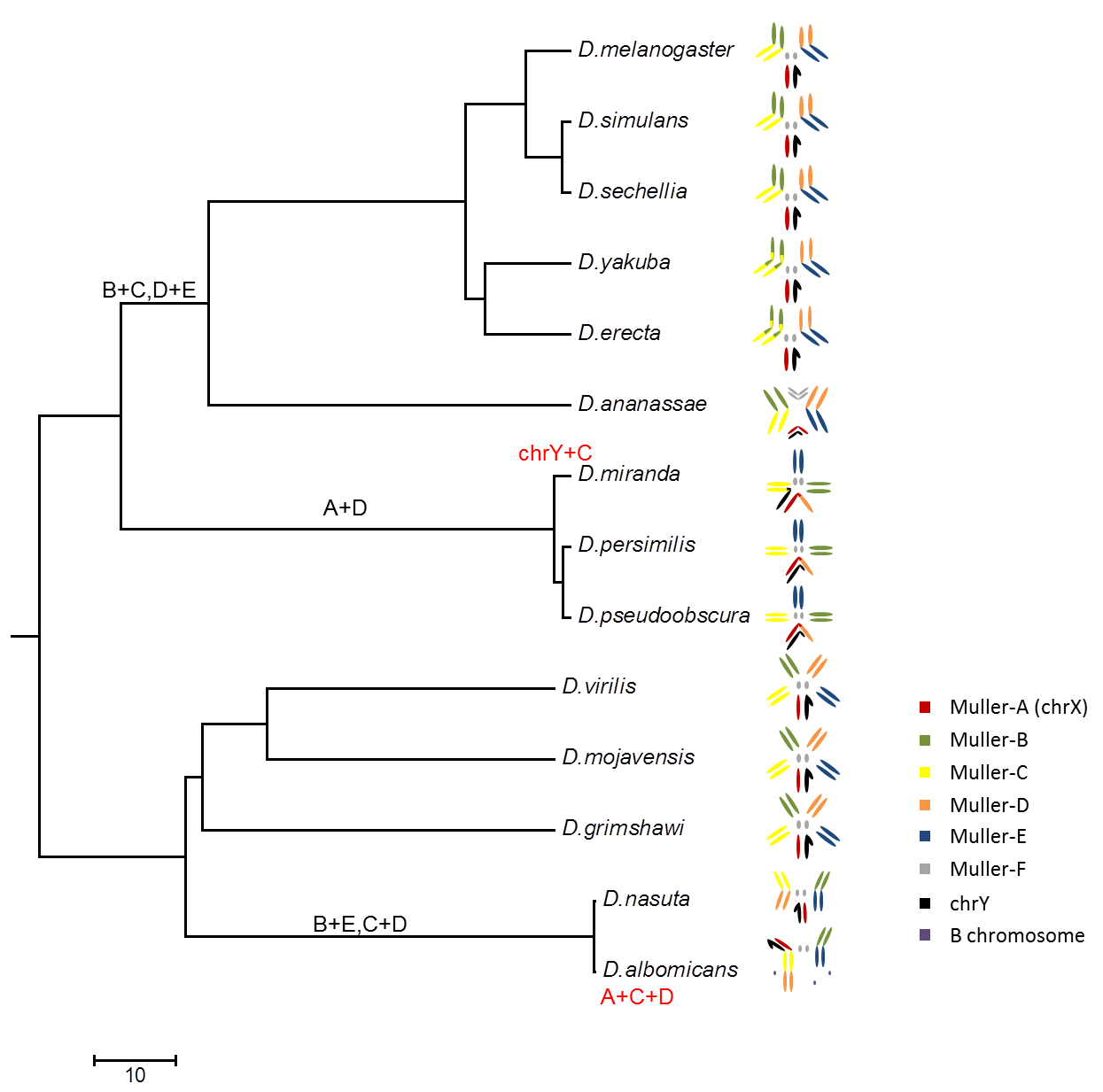
**

**Additional File 1: Figure S1. Karyotyp of *D. albomicans* and other *Drosophila* species.** Fusions of specific chromosomes (Muller’s elements) are shown along phylogenetic lineages. Modified from ref. [[1](#_ENREF_1)].

1. Chang TP, Tsai TH, Chang HY: **Fusions of Muller's elements during the chromosome evolution of Drosophila albomicans** *Zool Stud* 2008, **47**(5):574-584.
