# Supplementary material for: Deciphering neo-sex and B chromosome evolution by the draft genome of Drosophila albomicans
Source: BMC Genomics. 2012 Mar 22;13:109. doi: 10.1186/1471-2164-13-109 (PMC3353239; doi:10.1186/1471-2164-13-109)
Supplement: Additional file 2 — Figure S2 B chromosomes of D. albomicans. [file 1471-2164-13-109-S2.DOCX]

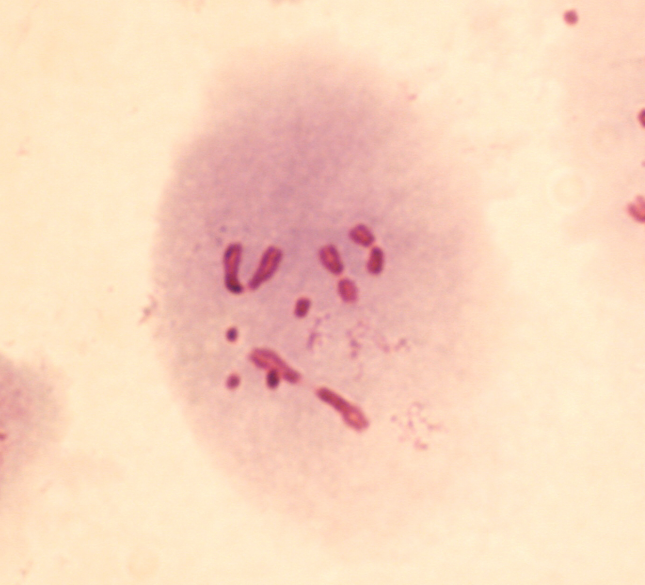

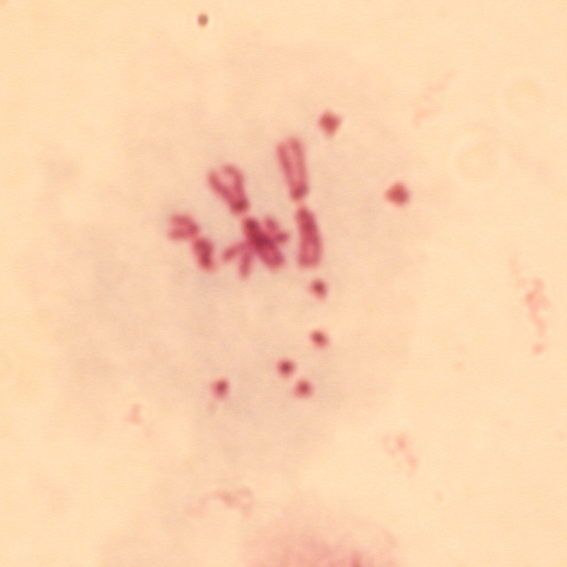

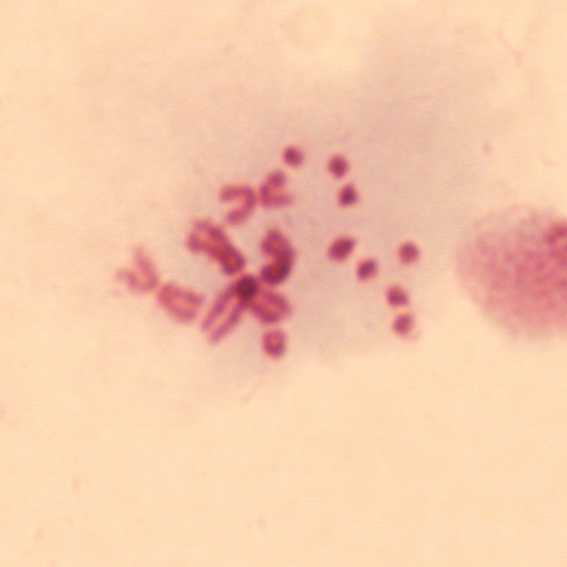

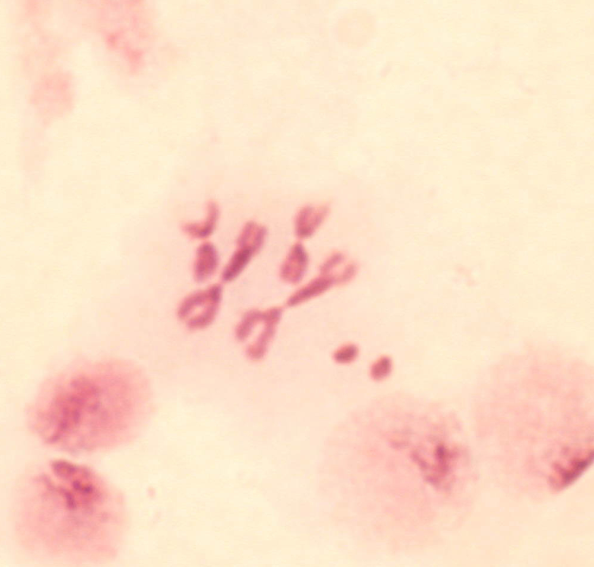


A

B

D

C

**Additional File 2: Figure S2. B chromosomes of *D. albomicans***

The figures show karyotyping results of *D. albomicans* strains containing different numbers of B chromosomes. B chromosome number usually differs between cells. A: 0B’s, B: 2B’s, C: 5B’s, D: 7B’s.
