# Supplementary material for: Deciphering neo-sex and B chromosome evolution by the draft genome of Drosophila albomicans
Source: BMC Genomics. 2012 Mar 22;13:109. doi: 10.1186/1471-2164-13-109 (PMC3353239; doi:10.1186/1471-2164-13-109)
Supplement: Additional file 3 — Table S1 Data summary of Illumina sequencing. [file 1471-2164-13-109-S3.DOCX]

**Additional File 3: Table S1 Data summary of Illumina sequencing**

| Sex | Library ID | Insert Size | Reads Production (Mb) | Read Length (bp) |
| --- | --- | --- | --- | --- |
| ♂ | DROzhqDAADBCAPE | 191 | 482.12 | 45 |
|  | DROzhqDAADBEAPE | 176 | 1805.4 | 45 |
|  | DROzhqDAADCDAPE | 233 | 7253.76 | 45 |
|  | DROzhqDAADGBAPE | 392 | 510 | 45 |
|  | DROzhqDABDBAAPE | 185 | 750.6 | 45 |
|  | DROzhqDABDDAAPE | 172 | 347.96 | 45 |
|  | DROzhqDABDJAAPE | 473 | 4664.24 | 45 |
|  | DROzhqDABDJBAPE | 491 | 4576.64 | 45 |
|  | DROzhqDABDLAAPE | 2000 | 327.98 | 45 |
|  | DROzhqDACDWAAPE | 2000 | 1515.78 | 75 |
|  | DROzhqDADDWAAPE | 2000 | 2704.82 | 75 |
|  | DROzhqDAADBCAPE | 191 | 482.12 | 45 |
| ♀ | L07037AADCAAPE | 211 | 1712.5 | 75 |
|  | L07037AADIAAPE | 504 | 4048.34 | 75 |
