# Supplementary material for: Deciphering neo-sex and B chromosome evolution by the draft genome of Drosophila albomicans
Source: BMC Genomics. 2012 Mar 22;13:109. doi: 10.1186/1471-2164-13-109 (PMC3353239; doi:10.1186/1471-2164-13-109)
Supplement: Additional file 4 — Table S2 Repetitive elements in the D. albomicans genome. [file 1471-2164-13-109-S4.DOCX]

**Additional File 4: Table S2 Repetitive elements in the *D. albomicans* genome**

| Repeat Type | Length | % Genome |
| --- | --- | --- |
| ARTEFACT: | 193 | 0.00% |
| DNA/hAT: | 11365 | 0.01% |
| DNA/Helitron: | 164849 | 0.08% |
| DNA/Maverick: | 3081 | 0.00% |
| DNA/MuDR: | 1709 | 0.00% |
| DNA/NOF: | 1803 | 0.00% |
| DNA/P: | 62482 | 0.03% |
| DNA/PiggyBac: | 1533 | 0.00% |
| DNA/TcMar-Mariner: | 37197 | 0.02% |
| DNA/TcMar-Tc1: | 31370 | 0.02% |
| DNA/Transib: | 51249 | 0.02% |
| LINE/CR1: | 16501 | 0.01% |
| LINE/I: | 10423 | 0.01% |
| LINE/Jockey: | 72566 | 0.04% |
| LINE/LOA: | 1741 | 0.00% |
| LINE/R1: | 28381 | 0.01% |
| LINE/R2: | 5286 | 0.00% |
| LINE/telomeric: | 262964 | 0.13% |
| Low_complexity: | 32929 | 0.02% |
| LTR/Copia: | 16195 | 0.01% |
| LTR/Gypsy: | 152074 | 0.07% |
| LTR/Pao: | 66907 | 0.03% |
| LTR: | 180 | 0.00% |
| Satellite: | 2864 | 0.00% |
| Simple_repeat: | 460838 | 0.22% |
| Unknown: | 9955709 | 4.82% |
| Simple Tandem Repeat | 13838299 | 6.59% |
