# Supplementary material for: Deciphering neo-sex and B chromosome evolution by the draft genome of Drosophila albomicans
Source: BMC Genomics. 2012 Mar 22;13:109. doi: 10.1186/1471-2164-13-109 (PMC3353239; doi:10.1186/1471-2164-13-109)
Supplement: Additional file 5 — Table S3 Sequence coverage comparison among chromosomes. [file 1471-2164-13-109-S5.DOCX]

**Additional File 5: Table S3 Sequencing coverage comparison among chromosomes**

| coverage/site | male | female |
| --- | --- | --- |
| chrX | 27.14**±**4.19 | 13.53±2.58 |
| chr2 | 44.73±11.21 | 10.82±3.08 |
| neo-sex(chr3) | 49.82±11.41 | 15.0±3.89 |
| chr4 | 39.98±15.89 | 9.12±3.70 |

We calculated average sequencing coverage (read count/base) and variances using male and female reads, respectively.
