# Supplementary material for: Deciphering neo-sex and B chromosome evolution by the draft genome of Drosophila albomicans
Source: BMC Genomics. 2012 Mar 22;13:109. doi: 10.1186/1471-2164-13-109 (PMC3353239; doi:10.1186/1471-2164-13-109)
Supplement: Additional file 6 — Figure S3 Short insertion (1-6 bp) densities along each chromosome. [file 1471-2164-13-109-S6.DOCX]

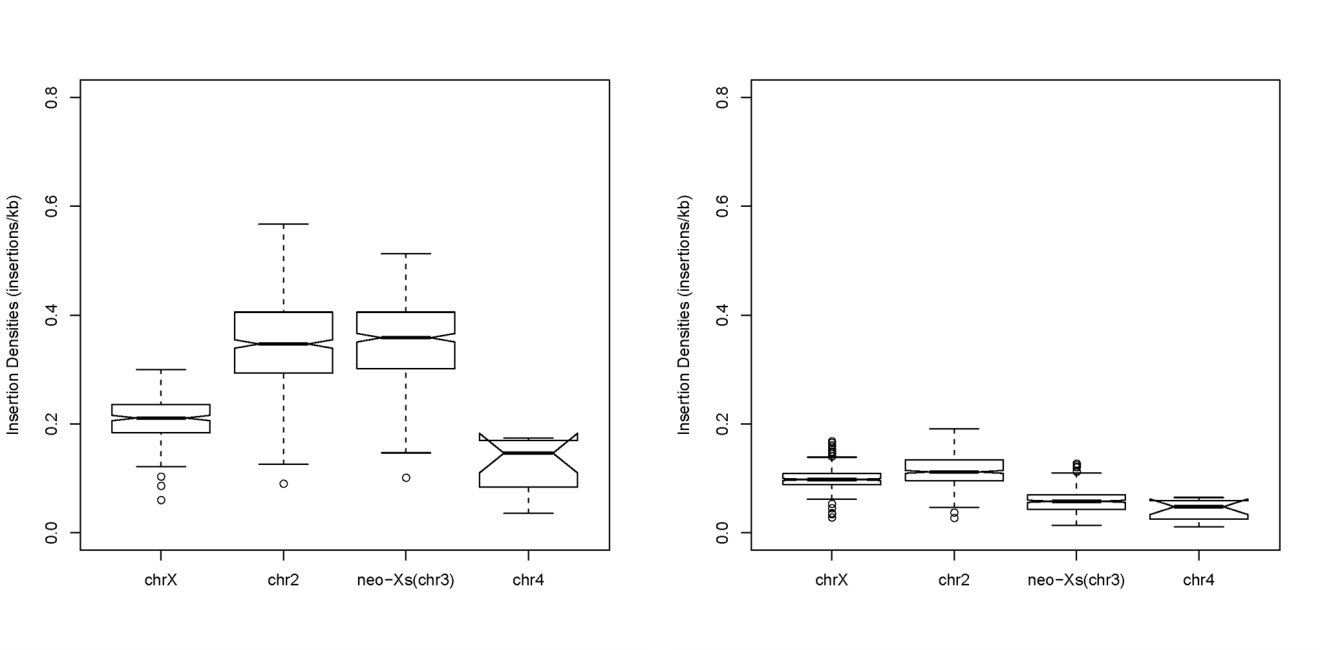


B.

A.

**Additional File 6: Figure S3 Short insertion (1-6bp) densities along each chromosome**

Densities of short insertions were calculated every 1Mb window with a step size of 100kb. (A) Short insertion densities using male reads. (B) Short insertion densities using female reads.
