# Supplementary material for: Deciphering neo-sex and B chromosome evolution by the draft genome of Drosophila albomicans
Source: BMC Genomics. 2012 Mar 22;13:109. doi: 10.1186/1471-2164-13-109 (PMC3353239; doi:10.1186/1471-2164-13-109)
Supplement: Additional file 7 — Figure S4 Short deletion densities along each chromosome. [file 1471-2164-13-109-S7.DOCX]

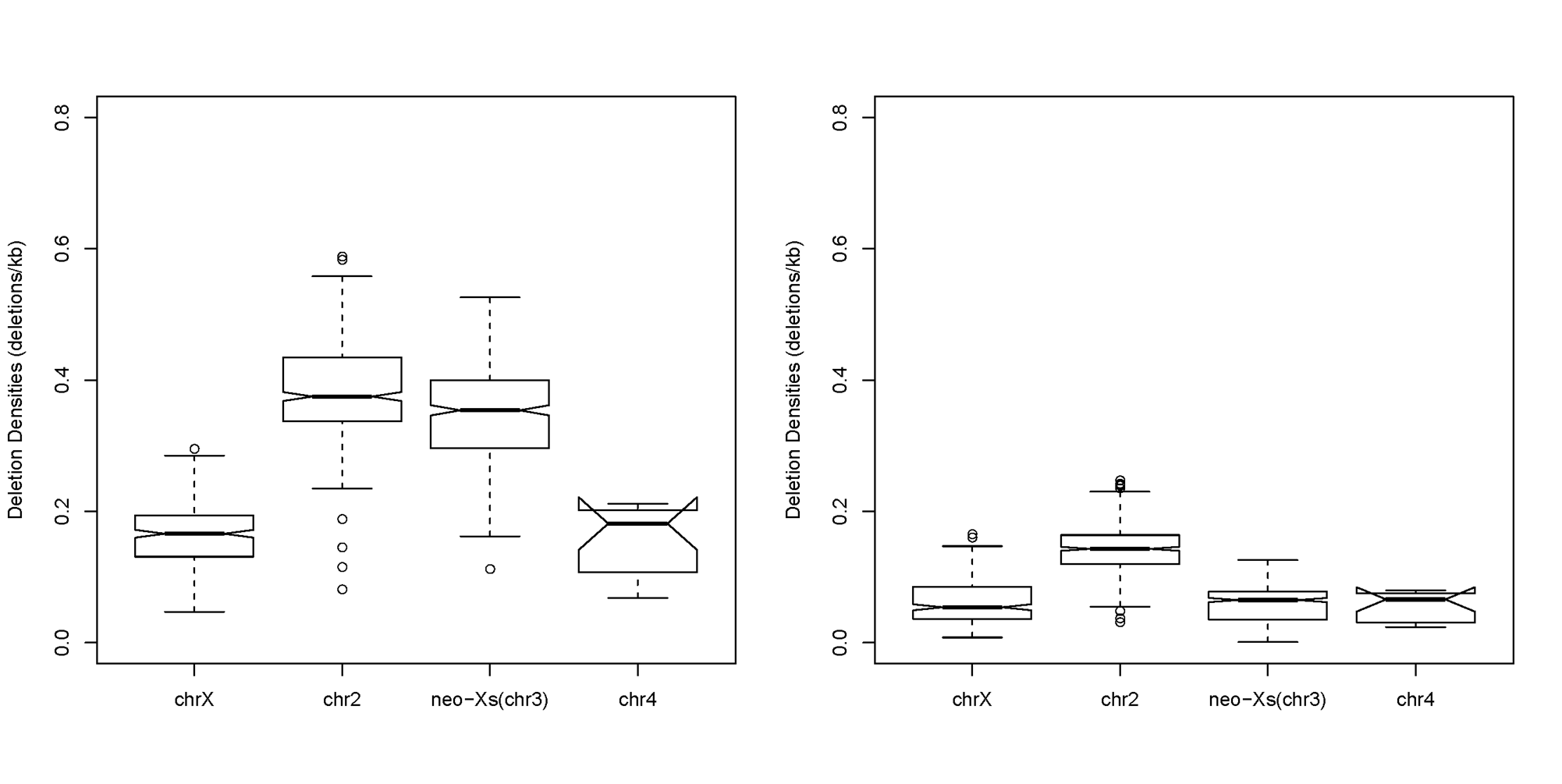


B.

A.

**Additional File 7:Figure S4 Short deletions (1-6bp) densities along each chromosome**

Densities of short deletions were calculated every 1Mb window with a step size of 100kb. (A) Short deletion densities using male reads. (B) Short deletion densities using female reads.
