# Supplementary material for: Deciphering neo-sex and B chromosome evolution by the draft genome of Drosophila albomicans
Source: BMC Genomics. 2012 Mar 22;13:109. doi: 10.1186/1471-2164-13-109 (PMC3353239; doi:10.1186/1471-2164-13-109)
Supplement: Additional file 8 — Table S4 SNP statistics for each chromosome. [file 1471-2164-13-109-S8.DOCX]

**Additional File 8: Table S4 SNP statistics for each chromosome**

|  | chrX | chr3 (neo-sex) | chr2 | chr4 |
| --- | --- | --- | --- | --- |
| Total SNP sites | 58252/21923 | 185197/35946 | 244099/120222 | 2615/432 |
| Ratio~1 SNP sites | 6294/3174 | 32746/3511 | 31869/17981 | 348/57 |
| Ratio~1 SNP % | 10.80/14.48 | 17.68/9.77 | 13.06/14.96 | 13.31/13.19 |
| Total Length | 28214712 | 44537821 | 51290274 | 1544049 |
| Ratio~1 SNP density (10^-3^) | 0. 2231/0.1125 | 0.7352/0.0788 | 0.6213/0.3536 | 0. 2254/0.0369 |

SNP statistics is shown as male/female. We defined SNPs with ratios between alternative nucleotides ranging between 0.75-1.25 as ratio~1 SNPs
