# Supplementary material for: Deciphering neo-sex and B chromosome evolution by the draft genome of Drosophila albomicans
Source: BMC Genomics. 2012 Mar 22;13:109. doi: 10.1186/1471-2164-13-109 (PMC3353239; doi:10.1186/1471-2164-13-109)
Supplement: Additional file 9 — Table S6 Structural variation identified by abnormally mapped male read pairs. [file 1471-2164-13-109-S9.DOCX]

**Additional File 9: Table S5 Codon usage on neo-sex chromosomes**

|  | ENC | CBI | FOP | CAI |
| --- | --- | --- | --- | --- |
| neo-X; mean | 50.57899 | 0.137846 | 0.449374 | 0.347642 |
| neo-Y; mean | 50.60759 | 0.137624 | 0.449231 | 0.347512 |
| neo-X; s.d. | 4.985582 | 0.111344 | 0.071179 | 0.068913 |
| neo-Y; s.d. | 4.988611 | 0.111567 | 0.071306 | 0.068916 |
| neo-X; median | 50.83 | 0.14 | 0.45 | 0.341 |
| neo-Y; median | 50.85 | 0.14 | 0.45 | 0.341 |

ENC: effective number of codons, ENC is 61 with random usage of codons, and 20 with extreme biased usage of codons; CBI: codon bias index, CBI is 0 with random usage of codons, and 1 with extreme biased usage of codons; FOP: frequency of optimal codons index and CAI: codon adaptation index, both of which are 0 without usage of optimal codons and 1 with a complete usage of optimal codons. S.d.: standard deviation.
