# Supplementary material for: Deciphering neo-sex and B chromosome evolution by the draft genome of Drosophila albomicans
Source: BMC Genomics. 2012 Mar 22;13:109. doi: 10.1186/1471-2164-13-109 (PMC3353239; doi:10.1186/1471-2164-13-109)
Supplement: Additional file 10 — Table S5 Codon usage on neo-sex chromosomes. [file 1471-2164-13-109-S10.DOCX]

Tandem Duplication

A

Dispersed Duplication

B

Deletion/Insertion

C

**Additional File 10: Figure S5. Identification of structural variation using mate-pair information.** Genomic regions involved in structural variation are shown as solid blue arrows and mate pairs are shown as blank blue/red arrows. (A) Tandem duplication; read pairs that normally map as ‘forward-reverse’ also show ‘reverse-forward’ mapping to the reference genome. (B) Dispersed duplication; reads located between/downstream of the duplicates show abnormal mapping to the reference genome. (C) Insertion/deletion; we define a difference of 3 times the insert size variance between mapping insert size and experimentally estimated size as a signature of an insertion/deletion event.
