# Supplementary material for: Deciphering neo-sex and B chromosome evolution by the draft genome of Drosophila albomicans
Source: BMC Genomics. 2012 Mar 22;13:109. doi: 10.1186/1471-2164-13-109 (PMC3353239; doi:10.1186/1471-2164-13-109)
Supplement: Additional file 11 — Figure S5 Identification of structural variation using mate-pair information. [file 1471-2164-13-109-S11.DOCX]

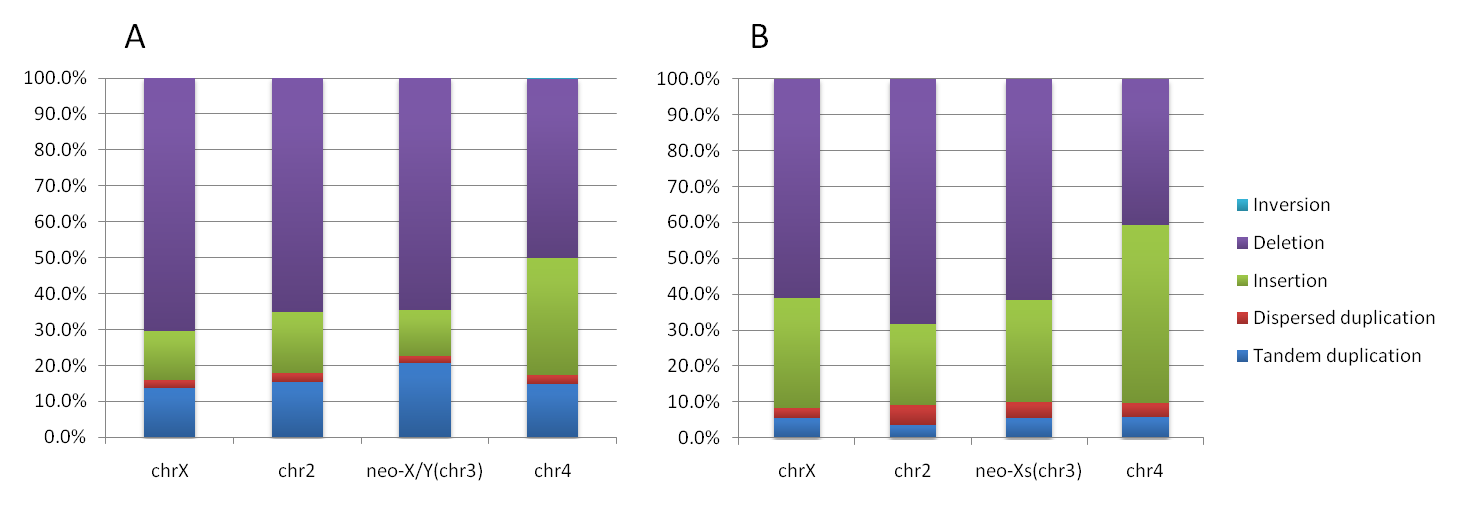


**Additional File 11: Figure S6. Fraction of different SV on Each Chromosome.**

(A) Structural variations identified using male reads. The proportion of tandem duplications is the highest on neo-sex chromosomes. (B) Structural variations identified using female reads. The proportion of tandem duplications on neo-sex chromosomes is similar to other chromosomes.
