# Supplementary material for: Deciphering neo-sex and B chromosome evolution by the draft genome of Drosophila albomicans
Source: BMC Genomics. 2012 Mar 22;13:109. doi: 10.1186/1471-2164-13-109 (PMC3353239; doi:10.1186/1471-2164-13-109)
Supplement: Additional file 12 — Figure S6 Fraction of different SV on Each Chromosome. [file 1471-2164-13-109-S12.DOCX]

**Additional File 12: Table S6 Structural variation identified by abnormally mapped male read pairs**

| male | chrX | chr2 | neo-sex | chr4 |
| --- | --- | --- | --- | --- |
| Insertion | 2102 | 7041 | 3671 | 473 |
| Deletion | 10679 | 26943 | 18602 | 728 |
| Tandem duplication | 2100 | 6350 | 5971 | 217 |
| Dispersed duplication | 300 | 1089 | 557 | 35 |
| Inversion | 2 | 17 | 10 | 3 |
| total | 15183 | 41440 | 28811 | 1456 |

This table lists the number of structural variation (SV) events on each chromosome inferred by mapping male reads. SVs were identified by abnormally mapped read pairs in terms of their orientations or size intervals. At least three read pairs were required to identify a SV event.
