# Supplementary material for: Deciphering neo-sex and B chromosome evolution by the draft genome of Drosophila albomicans
Source: BMC Genomics. 2012 Mar 22;13:109. doi: 10.1186/1471-2164-13-109 (PMC3353239; doi:10.1186/1471-2164-13-109)
Supplement: Additional file 13 — Figure S7. Proportions of dispersed duplications overlapping coding regions on each chromosome. [file 1471-2164-13-109-S13.DOCX]

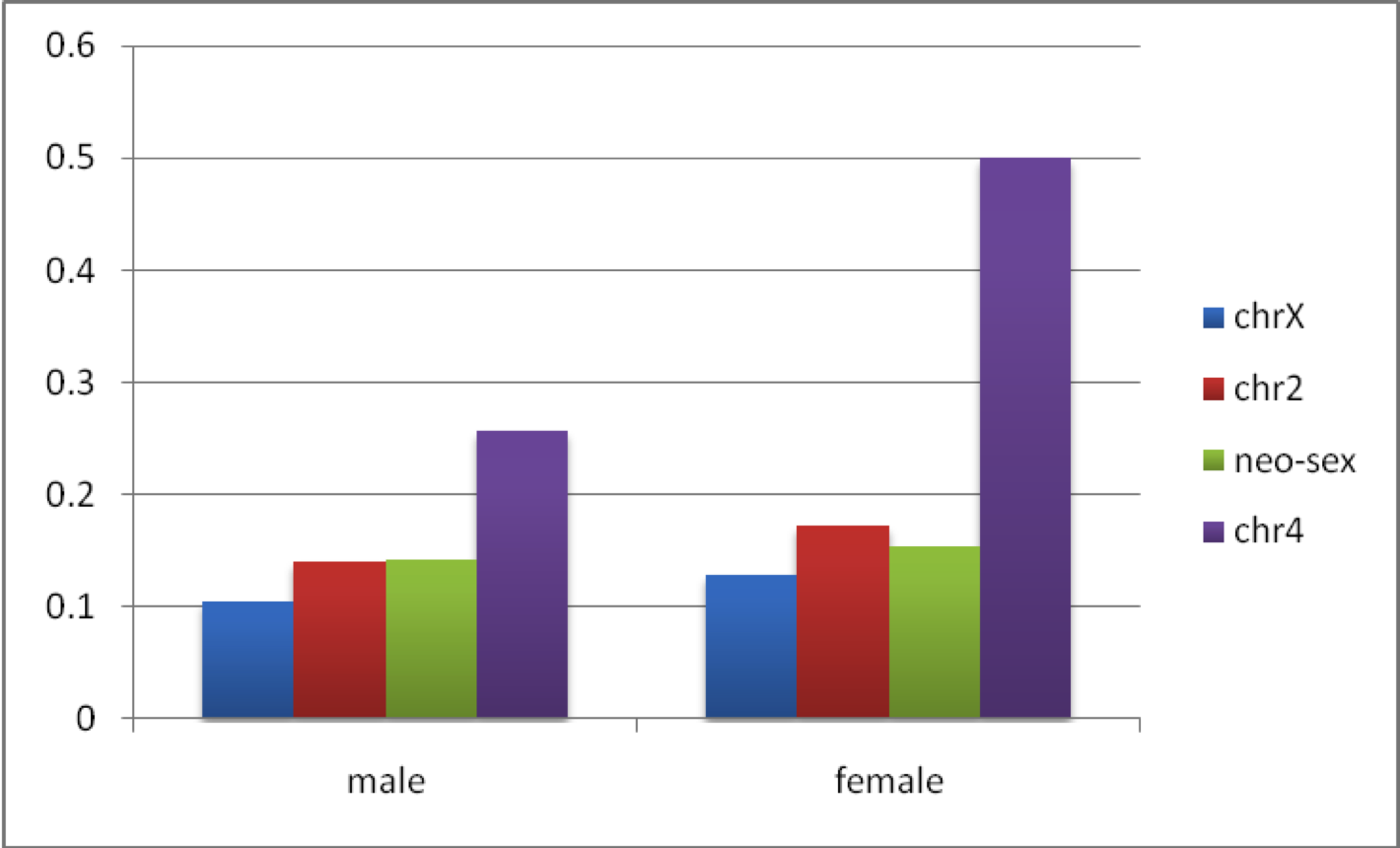


**Additional File 13: Figure S7. Proportions of dispersed duplications overlapping coding regions on each chromosome.**
