# Supplementary material for: Deciphering neo-sex and B chromosome evolution by the draft genome of Drosophila albomicans
Source: BMC Genomics. 2012 Mar 22;13:109. doi: 10.1186/1471-2164-13-109 (PMC3353239; doi:10.1186/1471-2164-13-109)
Supplement: Additional file 14 — Table S7 Statistics of scaffolds homologous to the B-specific probe. [file 1471-2164-13-109-S14.DOCX]

**Additional File 14: Table S7 Statistics of scaffolds homologous to the B-specific probe**

| Scaffolds | Length  (bp) | % aligned^#^ | Simple Tandem Repeats^*^ | *D. virilis*^§^ | *D. melanogaster* |
| --- | --- | --- | --- | --- | --- |
| S97157 | 252 | 58% (94%) | 0 | scaffold_13042 | chrX:3199190-3199230 |
| S94796 | 8266 | 65% (93%) | 5.95% | scaffold_13352 | chrX:13301418-13302555 |
|  |  |  |  | scaffold_12875 | chr2R:15154580-15154705 |
|  |  |  |  | scaffold_12963 | chr2L:5315423-5315644 |
| S86570 | 6739 | 56% (100%) | 28.5% | scaffold_12799 | chrX:11180546-11182019 |
| S51440 | 8499 | 75% (94%) | 20.86% | scaffold_12875 | chr2R:16553517-16553843 |
|  |  |  |  | scaffold_12928 | chrX:3512810-3512856 |

^#^The data shows the span length proportion (sequences identity) for the aligned region of the B-specific probe sequence with *D. albomicans* scaffolds.

*Proportion of simple tandem repeats occupying the whole length of the scaffolds was calculated using Tandem Repeat Finder [[1](#_ENREF_1)].

^§^ Orthologous sequences of the *D. albomicans* scaffolds in *D. virilis* and *D. melanogaster* were retrieved by blast and UCSC Genome Browser (http://genome.ucsc.edu/cgi-bin/hgGateway) to infer their locations.

1. Benson G: **Tandem repeats finder: a program to analyze DNA sequences**. *Nucleic Acids Res* 1999, **27**(2):573-580.
