# Supplementary material for: Deciphering neo-sex and B chromosome evolution by the draft genome of Drosophila albomicans
Source: BMC Genomics. 2012 Mar 22;13:109. doi: 10.1186/1471-2164-13-109 (PMC3353239; doi:10.1186/1471-2164-13-109)
Supplement: Additional file 15 — Table S8 Mapping result of candidate B-linked scaffolds with D. grimshawi and D. mojavensis. [file 1471-2164-13-109-S15.DOCX]

**Additional File 15: Table S8 Mapping result of candidate B-linked scaffolds with *D. grimshawi* and *D.*** ***mojavensis***

| *D. albomicans* | *D. mojavensis* | *D. melanogaster* | *D. grimshawi* | *D. melanogaster* |
| --- | --- | --- | --- | --- |
| S94796 | scaffold_6496 | chr2R:18054682-18055177 | scaffold_947 | chrX:18775500-18776620 |
| S94796 | scaffold_6680 | chrX:8323562-8324000 | scaffold_25041 | chrX:11179651-11184748 |
| S86570 | scaffold_6498 | chrX:11180310-11182034 | scaffold_24999 | chr2R:16563424-16564514 |
| S51440 | scaffold_6496 | chr2R:16563423-16564595 | scaffold_25041 | chrX:12581211-12581354 |
| S51440 | scaffold_6473 | chrX:12581186-12581353 | scaffold_24792 | chr2R:15153377-15153869 |

Candidate B-linked scaffolds were used as queries for blast searches against the genomes of *D. mojavensis* (droMoj2) and *D. grimshawi* (droGri1). The orthologous sequences of aligned regions in both species were then retrieved from UCSC Genome Browser (<http://genome.ucsc.edu/cgi-bin/hgGateway>) to localize their chromosomal information in *D. melanogaster*.
