# Supplementary material for: Deciphering neo-sex and B chromosome evolution by the draft genome of Drosophila albomicans
Source: BMC Genomics. 2012 Mar 22;13:109. doi: 10.1186/1471-2164-13-109 (PMC3353239; doi:10.1186/1471-2164-13-109)
Supplement: Additional file 16 — Table S9 Comparison of number of progenies in different D. albomicans strains. [file 1471-2164-13-109-S16.DOCX]

**Additional File 16: Table S9 Comparison of number of progenies in different *D. albomicans* strains**

| Strain | Investigated isofemale lines | Total progeny number | Average No. of progeny/pair | Average No. of females/pair | Average No. of males/pair | *P*-value  (ANOVA) |
| --- | --- | --- | --- | --- | --- | --- |
| 0B | 24 | 3768 | 157±7.05 | 75±2.83 | 82±4.65 | 0.0001 |
| 1-2Bs | 24 | 4704 | 196±3.34 | 88±5.66 | 107±3.95 | 0.0001 |
| >2Bs | 31 | 4557 | 147±3.57 | 68±2.82 | 79±3.50 | 0.001 |

Table modified from ref. [[1](#_ENREF_1)].

1. He LP, Ling FY, Zheng XZ, Wang W, Kuang RP: **The effect of B chromosome on the reproduction of Drosophila albomicans**. *Yi Chuan Xue Bao* 2000, **27**(2):114-120.
